# Preparation and Performance Study of MXene-Regulated Ethylene Glycol-Induced WO_3_ Film

**DOI:** 10.3390/mi15121486

**Published:** 2024-12-11

**Authors:** Yuqi Wang, Yong Liu, Minmin Wang, Wenjun Wu, Maofei Tian, Tao Zhu

**Affiliations:** 1 College of Materials and Metallurgy, Guizhou University, Guiyang 550025, China; 2 School of Energy and Power Engineering and State Key Laboratory of Coal and CBM Co-Mining, North University of China, Taiyuan 030051, China

**Keywords:** two-dimensional material, tungsten oxide, heterojunction, electrochromic

## Abstract

This study introduces the development of a W-M_1.0_ electrochromic film, characterized by a “coral”-like TiO_2_@WO_3_ heterostructure, synthesized via a hydrothermal process leveraging the inherent instability of MXene. The film showcases exceptional electrochromic performance, with a coloring response time of 2.8 s, a bleaching response time of 4.6 s, and a high coloring efficiency of 137.02 cm^2^C^−1^. It also demonstrates a superior light modulation ability of 73.83% at 1033 nm. Notably, the W-M_1.0_ film exhibits remarkable cyclic stability, retaining over 90% of its initial light modulation capacity after 4000 cycles, outperforming many existing electrochromic materials. The film’s enhanced performance is credited to its coral-like structure, which boosts the specific surface area and promotes ion transport, and the TiO_2_@WO_3_ heterojunctions, which enhance charge transfer and stabilize the material. Devices fabricated with the W-M_1.0_ film as the cathode and a PB film as the anode exhibit a seamless transition from dark blue to colorless, underscoring their potential for smart window and dynamic glass applications.

## 1. Introduction

Electrochromism is a phenomenon where electrochromic (EC) material undergoes electrolyte ion insertion/extraction under an applied electric field, leading to repeated changes in the chemical valence state and band structure, which in turn cause reversible alterations in its optical properties [1,2]. EC materials offer a multitude of advantages, such as intelligent color adjustment capabilities, high optical contrast, ease of control, low energy consumption, and robust stability [3,4,5,6]. Consequently, they have found broad applications in various domains, including smart windows, anti-glare rearview mirrors, electronic displays, memory devices, and wearable technology [7,8,9,10,11,12].

Inorganic materials have garnered significant attention due to their exceptional stability and well-established synthesis techniques. Tungsten oxide (WO_3_), the first inorganic material identified to exhibit electrochromic properties, has been extensively studied for optimizing its chromic behavior. It is well known that the electrochromic properties of WO_3_ are highly dependent on factors such as surface morphology, structure, composition, and crystallinity, all of which are closely linked to the film preparation process. Consequently, researchers focus on enhancing the performance of WO_3_ films by refining these preparation methods, including metal doping, designing nanostructures, and material compounding, which have proven effective in improving EC properties. However, these methods often lead to the formation of a dense WO_3_ membrane structure, which impedes the insertion and extraction of electrolyte ions and slows down ion migration. Additionally, this dense architecture decreases the reaction interface, limiting electrochemical reactivity and negatively impacting the response time and efficiency of electrochromic properties. During electrochromic processes, the materials expansion and contraction can create local stress concentrations, raising the likelihood of cracking and spalling, ultimately affecting cycle stability [13,14]. Therefore, simplifying the process to regulate the structure of WO_3_ films is crucial for advancing their practical applications.

The hydrothermal synthesis of WO_3_ films has emerged as a favored approach due to its low energy consumption, straightforward equipment requirements, and environmentally benign characteristics. This method facilitates film formation, with the introduction of a seed layer or the employment of self-seeding agents being instrumental in enhancing the deposition process. However, the incorporation of a seed layer can sometimes result in suboptimal interfacial bonding, which may compromise the film’s cyclic stability. In this context, the utilization of self-seeding agents for film formation presents considerable advantages, particularly in the fabrication of electrochromic WO_3_ films [15,16,17]. Studies have successfully demonstrated the fabrication of WO_3_ films using self-seeding agents, such as the creation of coral-like nanostructured films with enhanced cyclic stability through the use of glycerol as a self-seeding agent and ammonium sulfate as a capping agent [18]. Furthermore, ethylene glycol has been effectively utilized as a self-seeding agent to produce nest-like nanostructured WO_3_ films, which exhibit rapid response rates [15].

Herein, a high-performance W-M_x_ EC film (denotes X mL addition of MXene dispersion in the reaction) was successfully prepared using a simple hydrothermal method. Ethylene glycol was employed as a self-seeding agent to induce WO_3_ nucleation on the FTO substrate, followed by the introduction of a two-dimensional MXene material as a capping agent. Due to the unique two-dimensional sheet structure and unstable chemical feature of MXene, it is oxidized into TiO_2_ during the preparation process [19,20,21]. Therefore, this method is not only effective but also allows specific morphology to be controlled and also involves doping WO_3_ films through the introduction of TiO_2_ to enrich their energy band structure. As anticipated, the W-M_x_ films exhibited porous, coral-like nanostructures that provide ion transfer channels and abundant electrochemically active sites, and the TiO_2_-doped heterojunction film enriched the band structure that would promote the electronic transfer process. These favorable factors can effectively promote the EC process and lead to enhancements in EC performance, like response time, coloration efficiency, modulation capability, and cyclic stability.

## 2. Experimental Section

### 2.1. Materials

Sodium tungstate dihydrate (Na_2_WO_4_·2H_2_O), hydrochloric acid (HCl), hydrogen peroxide (H_2_O_2_), ethylene glycol (C_2_H_6_O_2_), propylene carbonate (C_4_H_6_O_3_), lithium fluoride (LiF), and lithium perchlorate (LiClO_4_) were procured from Aladdin Reagent Co., Ltd. (Shanghai, China), and used as received without further purification. Titanium aluminum carbide powder (TiAlC_2_) was sourced from Jilin Yiyi Technology Co., Ltd. (Changchun, China) and employed without additional purification. Fluorine-doped tin oxide (FTO) conductive glass substrates were obtained from Liaoning Preferred New Energy Technology Co., Ltd. (Yingkou, China), and were cut into dimensions of 0.8 × 4 cm^2^ prior to use. The substrates underwent ultrasonic cleaning in a sequence of acetone, isopropyl alcohol, deionized water, and ethanol for 30 min each, followed by immersion in ethanol for storage. Before experimental use, the glass substrates were dried using a nitrogen gun and subjected to ultraviolet ozone cleaning for 20 min to ensure surface sterility and removal of organic contaminants, thus preparing the substrates for subsequent experimental procedures.

### 2.2. Preparation of Precursor Solution

W-M_X_ films were synthesized via a one-step hydrothermal process. Initially, 3.29 g Na_2_WO_4_ 2H_2_O was dissolved in 30 mL of deionized water under continuous stirring to form a transparent solution. Subsequently, 3 M hydrochloric acid was added dropwise until no further precipitate formation occurred. The solution was then centrifuged at 5000 rpm to pellet the precipitate, which was washed with deionized water until neutral. The precipitate was redissolved in 30 mL hydrogen peroxide and diluted to 100 mL with deionized water at 60 °C to obtain the hydrothermal precursor solution. The etching of TiAlC_2_ powder resulted in the formation of accordion-like MXene sheets, as shown in Figure S1. The preparation of the MXene dispersion has been detailed in our previous publication [22].

### 2.3. Preparation of W-Mx Films

**Figure 1:**
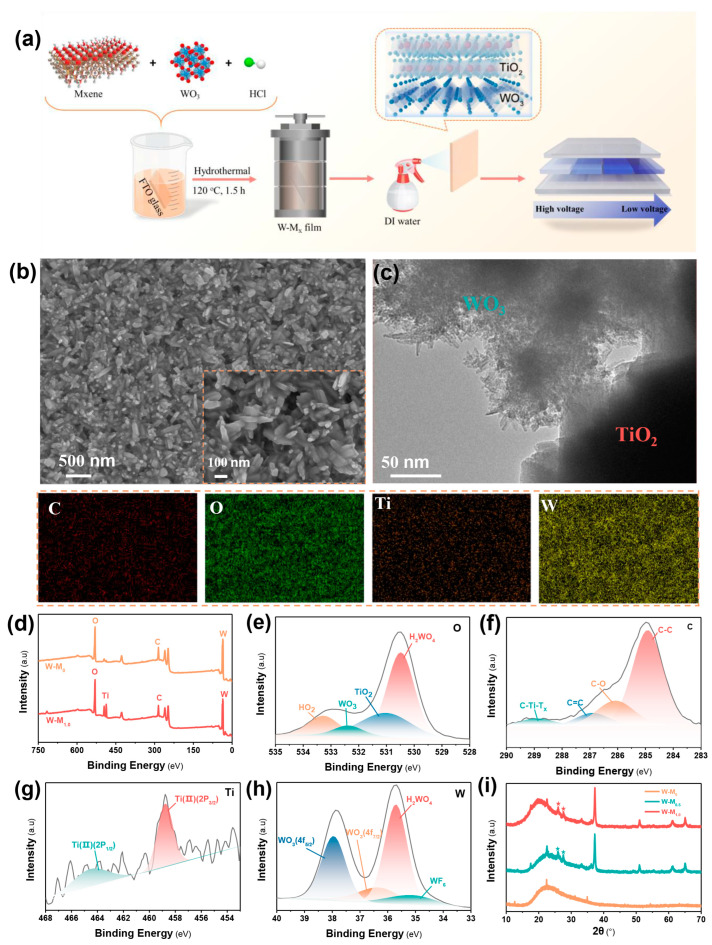
(**a**) Process flow diagram for the hydrothermal one-step synthesis of W-M_x_ films and a schematic of their color change under varying voltages. (**b**,**c**) SEM images and EDS-mapping and TEM images of the W-M_1.0_ films, respectively. (**d**–**i**) XPS and XRD characterization of the W-M_x_ films.

In the preparation of W-Mx films, 2 mL precursor solution, 0.3 mL 3M hydrochloric acid, 5 mL deionized water, and 1 mL ethylene glycol were combined with a specific volume of MXene dispersion liquid for ultrasonic homogenization. The resulting mixture was then transferred to the liner of a 25 mL Teflon-lined stainless-steel hydrothermal reaction vessel. A pristine fluorine-doped tin oxide (FTO) glass substrate, with the conductive surface facing downwards, was positioned within the liner. The liner was subsequently sealed within a high-pressure, explosion-proof reaction vessel, and the temperature of the blast drying oven was set to 120 °C for a duration of 2.5 h to facilitate the hydrothermal reaction, as shown in Figure 1a. The incorporation of varying quantities of the end-capping agent MXene served to modulate the film properties, thereby yielding the desired W-M_x_ films.

### 2.4. Fabrication of Electrochromic Device

Encapsulation of W-M_1.0_ EC devices was performed utilizing a methodical approach. PB (Prussian Blue) film and W-M_1.0_ film were selected as anode and cathode coloring materials, respectively, while PC/LiClO_4_ (1 M) was used as the electrolyte. The fabrication of W-M_1.0_ devices was followed by a series of performance tests to evaluate their functionality and efficiency.

### 2.5. Characterization

A comprehensive characterization of the composition and morphology of the W-M_x_ films was conducted using various analytical techniques. Scanning electron microscopy (SEM, Gemini 300, ZEISS, Oberkochen, Germany) and transmission electron microscopy (TEM, Tecnai F20, FEI, Hillsboro, OR, USA) were employed to examine the films’ microstructure and surface topography. Elemental analysis of the W-M_x_ films was performed using X-ray photoelectron spectroscopy (XPS, K-Alpha, Thermo Scientific, Waltham, MA, USA) and energy-dispersive X-ray spectroscopy (EDS) mapping, which was integrated with the SEM system (Gemini 300, ZEISS, Oberkochen, Germany). The crystallographic properties of the films were investigated through X-ray diffraction (XRD, X’Pert PRO MPD, PANalytical, Almelo, The Netherlands). The films’ and devices’ tuning ability, response time, coloration efficiency, and cycling stability were evaluated using a UV-Vis spectrophotometer (UV6300S, Mapada, Shanghai, China) in tandem with the electrochemical workstation (CHI 760E, Chenhua, Shanghai, China). The color coordinates (L*, a*, b*) of the W-M_x_ films were determined with a portable colorimeter (YS4510, 3nh, Shenzhen, China), providing quantitative data on the films’ color characteristics. These comprehensive analyses were essential for a thorough understanding of the W-M_x_ films’ properties and their potential applications in electrochromic devices.

## 3. Results and Discussion

### 3.1. Structural Characterization of W-M_X_ Films

To ascertain the morphological influence of MXene on W-M_x_ films, a comprehensive characterization was conducted using scanning electron microscopy (SEM), transmission electron microscopy (TEM), and X-ray photoelectron spectroscopy (XPS). Under identical process parameters, the W-M_x_ films synthesized via a one-step hydrothermal method are depicted in Figure 1a. In stark contrast to the nanofloral structure of the W-M_0_ membrane, the W-M_0.5_ and W-M_1.0_ membranes display a coral-like morphology (Figure 1b, Figures S2 and S3). During the formation of the W-M_0_ film, ethylene glycol induces the generation of sufficient nuclei, enabling WO_3_ crystals to grow unrestrained within the confined space. Upon contact, these crystals coalesce to form nanoflower-like clusters on the FTO surface. The dense structure of the W-M_0_ membrane results in a reduced specific surface area, consequently limiting the number of active sites available for electrochemical reactions.

The addition of MXene significantly enhances the control over the morphology of W-M_x_ films. In the W-M_0.5_ films, the presence of MXene as a capping agent restricts the growth of WO_3_ crystals within the confined space, preventing the formation of large nanoflower-like structures. Consequently, the W-M_0.5_ film exhibits a dispersed coral-like nanostructure. As the MXene content increases, the coral-like WO_3_ structure in the W-M_1.0_ membrane becomes even more dispersed. However, with further increments in MXene content, the growth of WO_3_ is increasingly inhibited, leading to a lack of distinct W-M_x_ film formation on the FTO surface.

The morphological analysis of the W-M_1.0_ films was conducted using TEM at multiple magnifications, providing a detailed examination of their structural features. The films were observed to possess a lamellar structure that is indicative of two-dimensional (2D) characteristics. This is attributed to the retention of the original 2D structure of MXene during its oxidative transformation into TiO_2_, as shown in Figure 1c. As a result, the W-M_1.0_ film exhibits a microstructural profile that is reminiscent of certain 2D materials, highlighting the influence of MXene on the film’s morphology. High-resolution TEM images, as presented in Figure S4, further elucidate the structural details of the W-M_1.0_ film. These images reveal the presence of lattice fringes measuring 0.34 nm in regions of high crystallinity and 0.24 nm in regions of low crystallinity. The lattice fringe at 0.34 nm corresponds to WO_3_, while the 0.24 nm lattice fringe corresponds to TiO_2_ with an anatase structure, in agreement with the literature [21,23]. The latter is a consequence of the oxidation of MXene by hydrogen peroxide (H_2_O_2_) during the hydrothermal reaction process, which is a critical step in the formation of the film [24,25,26].

To elucidate the role and distribution of MXene within the W-M_X_ film formation process, energy-dispersive X-ray spectroscopy mapping (EDS-Mapping) was employed to analyze the elemental composition on the surface of the W-M_x_ films. The W-M_0_ film, which consists solely of WO_3_, exhibits a nanoflower structure with the distribution of W and O elements closely matching this morphology, as shown in Figure S5. The EDS-Mapping results for the W-M_1.0_ films, as shown in Figure 1b, reveal the concurrent presence of W, O, C, and Ti elements. The elements W and O are predominantly derived from WO_3_, while C is contributed by both MXene and ethylene glycol. The Ti element, which is a signature component of MXene, is uniformly dispersed throughout the W-M_1.0_ film, indicating the thorough incorporation of MXene within the film’s structure. These findings suggest that ethylene glycol plays a crucial role in the hydrothermal reaction by facilitating the nucleation and growth of WO_3_ grains on the FTO surface through the formation of “bridge bonds”. MXene, on the other hand, plays a dual role in film formation and structural regulation, ultimately existing within the film as anatase TiO_2_ post-reaction.

The elements in W-M_x_ films were characterized by XPS, revealing peaks at 36.1, 530.1, and 285.1 eV for W, O, and C, respectively. The W and O elements originate from WO_3_, while C and O elements from the crystallization agent ethylene glycol are only present in MXene, which also contains Ti elements [27,28]. Compared to the W-M_0_ film, the W-M_1.0_ film exhibited a higher detection of Ti elements (Figure 1d) [29,30]. This confirms that MXene is instrumental in regulating the morphology and composition of W-M_1.0_ films during their formation.

The XPS W4f characteristic spectrum of the W-M_1.0_ film shows peaks at 37.98, 36.38, 35.68, and 35.08 eV, corresponding to the characteristic peaks of WO_3_(4f_7/2_), WO_3_(4f_5/2_), H_2_WO_4_, and WF_6_, respectively, as shown in Figure 1h. The XPS O 1s characteristic spectrum, with peaks at 533.28, 532.38, 531.08, and 530.48 eV, corresponds to H_2_O, WO_3_, TiO_2_, and H_2_WO_4_, respectively, as shown in Figure 1e. The XPS C 1s characteristic spectrum, with peaks at 288.88, 286.98, and 284.98 eV, corresponds to C-Ti-T_x_, C=O, C-O, and C-C bonds, respectively, as shown in Figure 1f. The XPS Ti 2p spectrum, with peaks at 464.38 and 458.78 eV, corresponds to the Ti (II)(2p_1/2_) and Ti(II)(2p_3/2_) characteristic peaks, respectively, as shown in Figure 1g. XPS analysis confirms the presence of TiO_2_ in the anatase structure in the W-M_1.0_ film following the one-step hydrothermal reaction with MXene.

As shown in Figure 1i, the XRD patterns of W-M_x_ and W-M_1.0_ films display characteristic peaks for FTO (indium tin oxide) at 37.28°, 50.96°, 61°, and 65.07° [31]. Additionally, TiO_2_ characteristic peaks are observed at 25.93° and 27.54° [32,33]. In contrast, W-M_0_ films do not show prominent FTO or TiO_2_ peaks. The dense structure and significant thickness of the films make it challenging to detect FTO signals in XRD tests (Figure S6), with the results primarily highlighting tungsten oxide characteristic peaks. This confirms that MXene was oxidized to anatase TiO_2_ under acidic conditions during the preparation of W-M_x_ films, contributing to the adjustment of morphology and construction of the films.

### 3.2. Electrochemical Properties of the W-M_x_ Film

**Figure 2:**
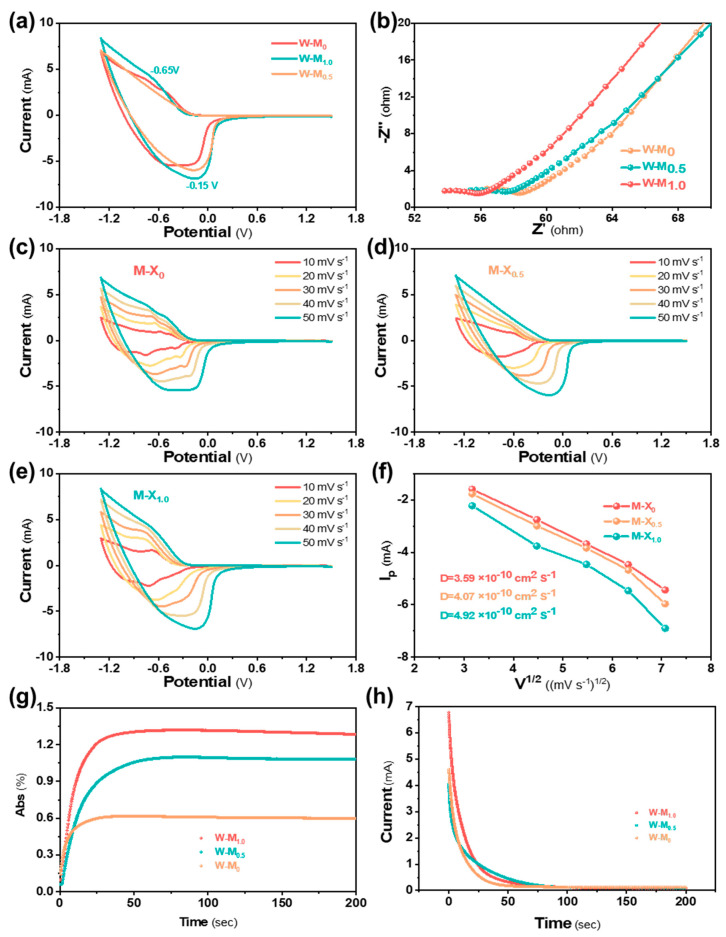
(**a**) The CV curves of W-M_X_ films. (**b**) The Nyquist plots of the W-M_x_ films. (**c**–**e**) The CV curves of W-M_X_ films at different scanning rates. (**f**) Current density changes with the square root of the scanning rates. (**g**) The relationship between absorption and time change in W-M_x_ films. (**h**) The relationship between current and time change in W-M_x_ films.

The cyclic voltammetry curves of W-M_x_ films were studied, as shown in Figure 2a. During testing, W^6+^ in the W-M_x_ film undergoes continuous reduction to W^5+^. The current peaks when the reduction rate of W^6+^ is at its highest before gradually declining. As the scanning speed increases, the electrochemical reactions occurring on the electrode surface can reach a stable state more rapidly, leading to a larger current response and consequently increasing the area of the curve, as shown in Figure 2c–e. Notably, the reduction peak of the W-M_1.0_ film is shifted compared to that of the W-M_0_ film. The shift can be attributed to the introduction of MXene, which causes distortion of the WO_3_ lattice and alters the characteristics of the film interface. These changes lead to modifications in the energy barrier of the charge transfer process, thereby impacting the reduction reaction potential.

The electrochemical impedance of W-M_0_ and W-M_1.0_ films was analyzed via Nyquist curves in Figure 2b. Both films exhibit similar arc radii in the high-frequency region, indicating consistent system resistance, electrode capacitance, and polarization resistance. However, the low-frequency region shows significant slope differences due to varying ion diffusion impedances. The loose structure of W-M_1.0_ film facilitates ion transport, while the TiO_2_@WO_3_ heterostructure enhances charge transfer. Conversely, the dense structure of W-M_0_ nanoparticles and single energy bands hinders ion transport.

When a coloration voltage is applied to W-M_x_ films, the transmittance and current of the film change over time, as shown in Figure 2g,h, with the absorption increasing rapidly at the initial stage. When a bias voltage is applied, the current profile exhibits a rapid response followed by a sharp decrease, indicating an efficient electrochemical reaction process controlled by ion diffusion. The electrochromic process of W-M_x_ films is controlled by the diffusion of electrolyte ions during the application of the colored voltage. In this process, Li^+^ ions are repeatedly intercalated and deintercalated in the film, altering the valence state of the W element and causing a reversible redox reaction in the material system. The TiO_2_@WO_3_ heterostructure in the W-M_1.0_ film deforms at the grain boundaries, leading to spatial migration. The diffusion coefficient of Li^+^ in the W-M_0_, W-M_0.5_, and W-M_1.0_ films can be calculated using the Randles-Sevcik Equation (1) [34]

(1) $$i=2.69\times 10^{5}\times n^{3/2}\times C_{0}\times A\times D^{1/2}\times v^{1/2}$$

where *i* is the peak current (mA), *D* is the diffusion coefficient (cm^2^·s^−1^), *A* is the working electrode area (cm^2^), *n* is the number of electrons transferred, *C_0_* is the concentration of active ions (mol·cm^−3^), and *v* is the scan rate in cyclic voltammetry (mV·s^−1^).

The ion diffusion coefficients of the W-M_0,_ W-M_0.5_, and W-M_1.0_ films are 3.59 × 10^−10^, 4.07 × 10^−10^, and 4.92 × 10^−10^ cm^2^ S^−1^, respectively. The different chemical compositions and lattice structures of TiO_2_ and WO_3_ in the heterostructure induce lattice distortion, enhancing the electrochemical reactivity of the materials. Additionally, the varied energy band arrangement reduces the charge transfer barrier, making it easier for charges to transfer at the interface. As the MXene content increases, the lattice distortion and energy band changes become more pronounced, leading to an increase in the ion diffusion coefficient of the W-M_x_ films.

### 3.3. Electrochromic Properties of W-M_x_ Films

**Figure 3:**
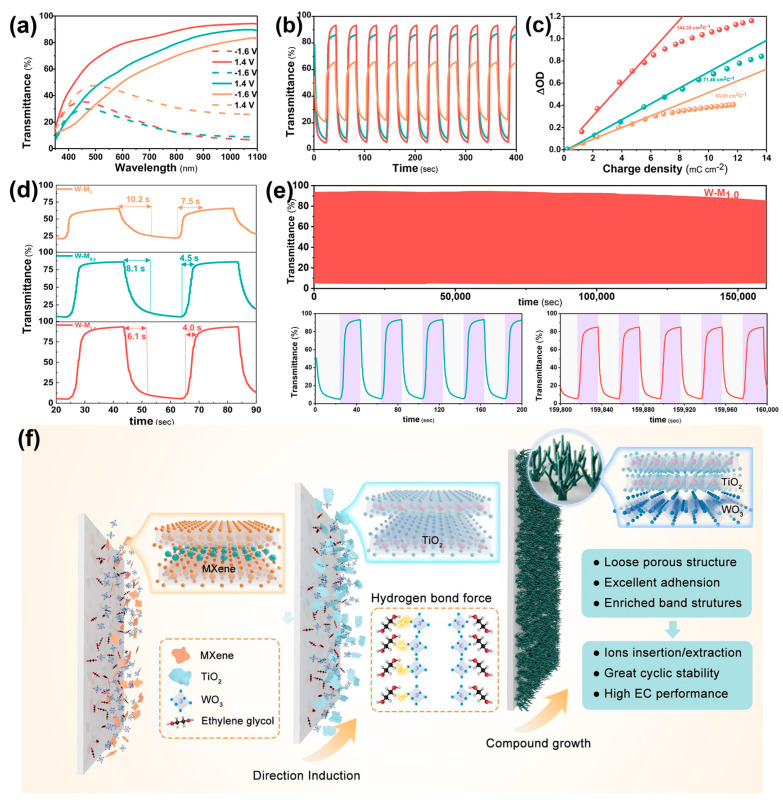
EC performance of W-M_x_ films: (**a**) Transmittance curves of W-M_0_ (yellow), W-M_0.5_ (green), and W-M_1.0_ (red) films at different voltages. (**b**) Optical modulation of W-M_x_ films at 1033 nm. (**c**) Coloration efficiency of W-M_x_ film. (**d**) Response time of W-M_x_ films. (**e**) Long-term stability of W-M_1.0_ films. (**f**) Schematic illustration of the fabrication process of the TiO_2_@WO_3_ heterostructure.

The addition of MXene significantly enhanced the electrochemical properties of the W-M_x_ films, leading to further exploration of their electrochromic behavior. As shown in Figure 3a, the optical transmission curves of the W-M_x_ films show distinct differences when the applied voltage varies from −1.6 V to 1.4 V, particularly in the near-infrared region, where the films exhibit excellent modulation capability (ΔT). Notably, the W-M_1.0_ film displays the largest modulation range, while the W-M_0_ film shows the smallest. At a voltage of −1.6 V, the W-M_1.0_ film is in a colored state, with a transmittance of only 6.69% at 1033 nm. When the voltage is increased to 1.4 V, the W-M_1.0_ film fades, and its transmittance rises to 94.30%, resulting in a ΔT value of 87.61% at 1033 nm. In comparison, the ΔT values for the W-M_0.5_ and W-M_0_ films at 1033 nm are 77.92% and 44.87%, respectively, as shown in Figure 3b.

The CIELAB color coordinates (L*, a*, b*) of the W-M_1.0_ film in both its colored and faded states were quantitatively determined using a portable color difference meter. In the colored state (−1.6 V), the color coordinate values are (50.48, −3.70, −18.04), while in the faded state (1.4 V), the values shift to (74.53, 0.57, 0.19). The corresponding color coordinates and optical photographs for these different states are shown in Figure S7, and the switching process is shown in SMOV1.

Response time is a critical factor in assessing the performance of electrochromic materials, typically defined as the time required to achieve 90% of the maximum modulation capacity. As shown in Figure 3d, the W-M_1.0_ film exhibited a coloring time of 6.1 s and a fading time of 4.0 s. In comparison, the W-M_0.5_ film had a coloring time of 8.1 s and a fading time of 4.5 s, while the W-M_0_ film showed a coloring time of 10.2 s and a fading time of 7.5 s.

Coloring efficiency (CE) is a crucial parameter for assessing the speed of optical modulation in electrochromic materials. It is defined as the ratio of the change in optical absorption at a specific wavelength to the amount of charge gained or lost per unit area. This can be calculated using the following Equation (2) [35]:

(2) $$\mathrm{CE}=\frac{\Delta \mathrm{OD}}{Q_{d}}=\frac{\log(T/T_{c})}{Q_{d}}$$

where T represents the transmittance at different time points, T_c_ is the transmittance of the colored state, and Q_d_ is the amount of charge injected or withdrawn per unit area. The ΔOD and Q_d_ at different time points were plotted in a scatter plot and subjected to linear fitting to determine the coloring efficiency of the W-M_0_, W-M_0.5_, and W-M_1.0_ films, which were calculated to be 53.03, 71.46, and 144.35 cm^2^ C^−1^, respectively, as shown in Figure 3c.

The results indicate that with increased MXene content, the response time of the films is reduced and the coloring efficiency is improved. The enhancement is attributed to the loose coral-like structure of the W-M_1.0_ film, which facilitates electrolyte ion transport, in contrast to the dense nanoflower structure of the W-M_0_ film that impedes ion transport. The coral-like morphology of the W-M_1.0_ film offers more reaction sites for the electrochromic process, thereby accelerating the reaction rate. Additionally, the TiO_2_ formed from MXene introduction integrates effectively with WO_3_, enhancing electron transfer during the electrochemical reaction and thus increasing the electrochromic reaction rate, as shown in Figure 3f.

Cyclic stability is a crucial index for evaluating their performance. To assess electrochromic film stability, step voltages of −1.6 V and 1.4 V were applied to W-M_x_ films for several cycles, with each step lasting 20 s. The change in transmittance at a wavelength of 1033 nm was recorded in real-time, as shown in Figure 3e and Figure S8. Initially, the optical regulation ability of the W-M_0_ film slightly decreased. This decrease occurred during the early cycles as the film established its electrolyte ion transport channels, causing minor structural collapse. Once these channels were properly formed, the film’s optical regulation ability stabilized. However, after 2000 cycles (80,000 s), the ΔT value of the W-M_0_ film diminished by 19.21%, indicating a relatively quick decline in its optical regulation ability and poorer cyclic stability.

In contrast, the W-M_1.0_ film demonstrated the least attenuation of regulatory capacity during cyclic testing. As shown in Figure 3f, this can be attributed to the heterostructure formed between TiO_2_ and WO_3_ that creates a better interface that enhances charge transfer while reducing interface defects, thereby improving stability. The dispersed coral-like structure helps to increase the specific surface area of the film, contributing to higher ion transport rates and larger electrochemical reaction interfaces. Concurrently, the potential damage to the film’s structure, which can be caused by lattice expansion and contraction during the ion embedding and deembedding processes, is mitigated. Additionally, the enriched band structure of the TiO_2_/WO_3_ heterojunction improves the conductivity and high carrier concentration to promote the electronic transfer to facilitate electrochemical reaction rates and enhance the EC performance. Consequently, the W-M_1.0_ film exhibited superior cycle stability, with only a 9.89% reduction in its photomodulation capacity after 4000 cycles (160,000 s). The regulation capacity of the W-M_1.0_ film reached 87.61% at a wavelength of 1033 nm, showcasing better cyclic stability compared to most previously reported [36,37,38,39].

In the field of electrochromic materials, many materials perform well in certain aspects, but achieving a balance between optical contrast, response speed, and optical transmittance remains challenging. According to the comparison data in Table S1, while some modified WO_3_ films and other electrochromic materials excel in optical contrast or response speed, their coloration efficiency and cycling stability are generally inferior to those of the W-M_1.0_ film, often significantly lower, which limits the further development of these materials [40,41,42]. In contrast, the W-M_1.0_ film demonstrates fast response, remarkable coloration efficiency, and excellent cycling stability, offering unique advantages for electrochromic applications. For instance, it can achieve rapid color switching within a short period and offers a wide range of optical modulation, making it highly attractive for applications such as smart windows, optical blinds, and display devices. Additionally, the exceptional cycling stability of W-M_1.0_ ensures that it can maintain stable electrochromic performance over extended periods of use, making it an ideal material for wearable devices, portable electronics, e-readers, and flexible displays. In these devices, the electrochromic effect not only provides an energy-efficient alternative to traditional displays but also meets complex optical modulation requirements.

### 3.4. Fabrication of Electrochromic Device

**Figure 4:**
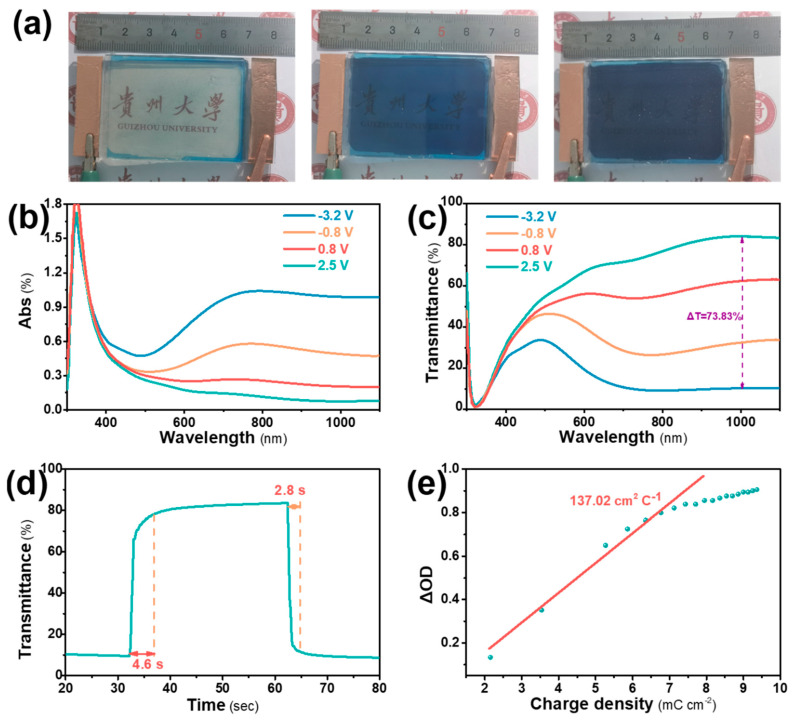
The ECD performances of W-M_1.0_ film are (**a**) optical images, (**b**) absorption curves, (**c**) transmittance curves, (**d**) response time, and (**e**) coloration efficiency.

To further assess the electrochromic performance and practical application potential of the W-M_1.0_ film, PB film was chosen as the anode electrochromic material and W-M_1.0_ as the cathode. PC/LiClO_4_ (1 M) was used as the electrolyte, and devices measuring 6.0 × 4.0 cm^2^ were fabricated. When a voltage of −3.2 V was applied, the EC device based on the W-M_1.0_ film displayed a dark blue color, while at 2.5 V, it turned colorless, as shown in Figure 4a. As the voltage was gradually increased from −3.2 V to 2.5 V, the color of the electrochromic device transitioned smoothly, accompanied by a decrease in absorption rate, as shown in Figure 4b. The device achieved a significant optical contrast of 73.83% at 1033 nm within the voltage range of −3.2 to 2.5 V, as shown in Figure 4c and SMOV2. The response times were measured under a −3.2/2.5 V step voltage, revealing a coloration time of 2.8 s and a bleached time of 4.6 s, as shown in Figure 4d. When a constant colored voltage of −3.2 V was applied, the device transitioned from colorless to dark blue, with changes in optical transmittance recorded at 1033 nm, as shown in Figure 4e. The calculated coloration efficiency of the device was 137.02 cm^2^C^−1^. These findings demonstrate that the W-M_1.0_ device exhibits excellent electrochromic properties, underscoring its potential for practical applications.

## 4. Conclusions

In summary, the optimal process for preparing WO_3_ EC membranes via hydrothermal methods involves using ethylene glycol as a self-seeding agent, two-dimensional MXene as a capping agent, and producing W-M_x_ films under acidic conditions. During the preparation, MXene is oxidized into a rutile-type TiO_2_ structure, which regulates the overall film structure and forms a porous coral-like heterostructure through mutual doping with WO_3_. The interfacial bonding, unique band structure, and lattice distortion within the heterostructure enhance the electrochromic properties of the W-M_x_ films. The preparation process for the W-M_1.0_ film is straightforward, featuring a low redox voltage window, a fast ion migration rate (4.92 × 10^−10^ cm^2^/S), short response times (colored time of 6.1 s, bleached time of 4.0 s), high coloration efficiency (144.35 cm^2^/C), and excellent cyclic stability (ΔT decays only 9.89% after 4000 cycles at 1033 nm). Furthermore, the EC device based on W-M_1.0_ film, when packaged and evaluated, demonstrated good modulation capability (73.83% at 1033 nm), short response times (colored time of 2.8 s, bleached time of 4.6 s), and high coloration efficiency (137.02 cm^2^/C). Thus, the EC film produced by this method holds significant practical application value.

## Data Availability

The original contributions presented in the study are included in the article; further inquiries can be directed to the corresponding authors.

## Supplementary Materials

The following supporting information can be downloaded at: https://www.mdpi.com/article/10.3390/mi15121486/s1, Figure S1. TiC_2_T_x_ sheet after etching. Figure S2. SEM image of the W-M_0_ film. Figure S3. SEM image of the W-M_0.5_ film. Figure S4. HR-TEM images of the W-M_1.0_ films. Figure S5. EDS-Mapping of the W-M_0_ film. Figure S6. The thickness of (a) W-M_0_ film, (b) W-M_0.5_ film and W-M_1.0_ film. Figure S7. Optical photos and color values of W-M_1.0_ film under different voltages. Figure S8. Long-term stability of (a) W-M_0_ films and (b) W-M_0.5_ films. Table S1. Comparison of t_c_, t_b_, ΔT%, CE and cycling stability of W-M_1.0_ films with other electrochromic materials.
