# Supplementary material for: Preparation and Performance Study of MXene-Regulated Ethylene Glycol-Induced WO3 Film
Source: Micromachines (Basel). 2024 Dec 11;15(12):1486. doi: 10.3390/mi15121486 (PMC11728441; doi:10.3390/mi15121486)
Supplement: Supplementary file 1 [file micromachines-15-01486-s001.zip › micromachines-3360869-supplementary.pdf]

Preparation and Performance Study of MXene-Regulated Ethylene Glycol-Induced WO_3 Film

Yuqi Wang[1†], Yong Liu[1†], Minmin Wang[2], Wenjun Wu[1], Maofei Tian[1], Tao Zhu[1*]

[1]College of Materials and Metallurgy, Guizhou University, Guiyang 550025, China;

[2]School of Energy and Power Engineering & State Key Laboratory of Coal and CBM Co-Mining, North University of China, Taiyuan 030051, PR China

Email: ivzhutao@126.com

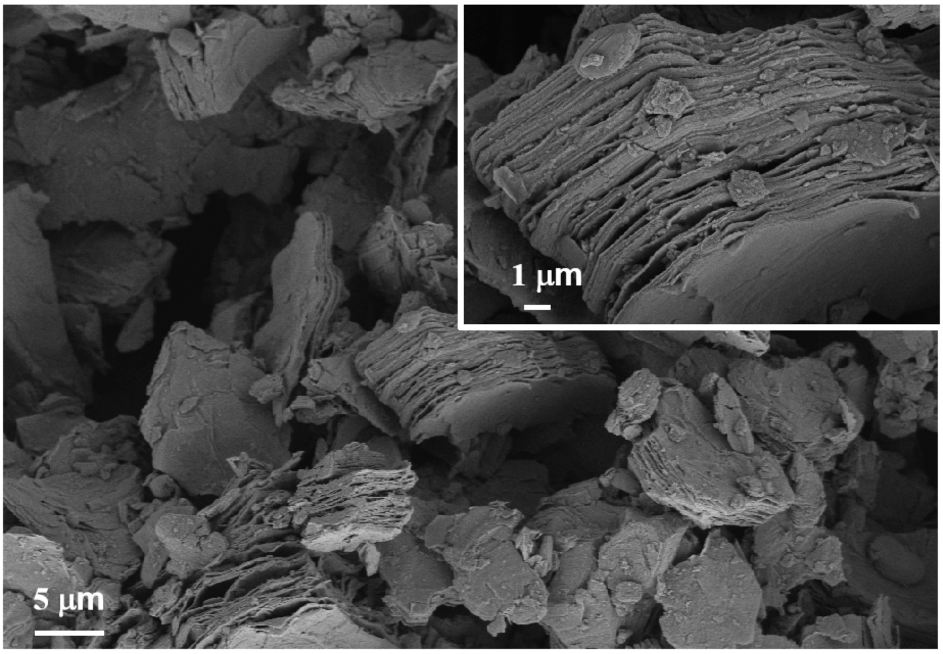

Figure S1 TiC_2T_x sheet after etching

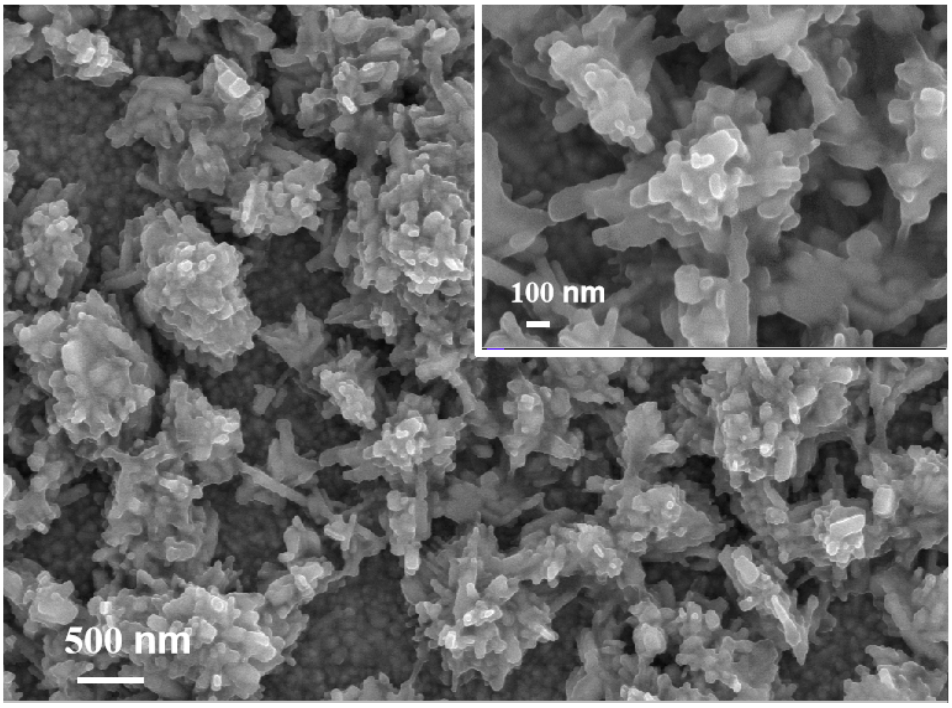

Figure S2 SEM image of the W-M_0 film

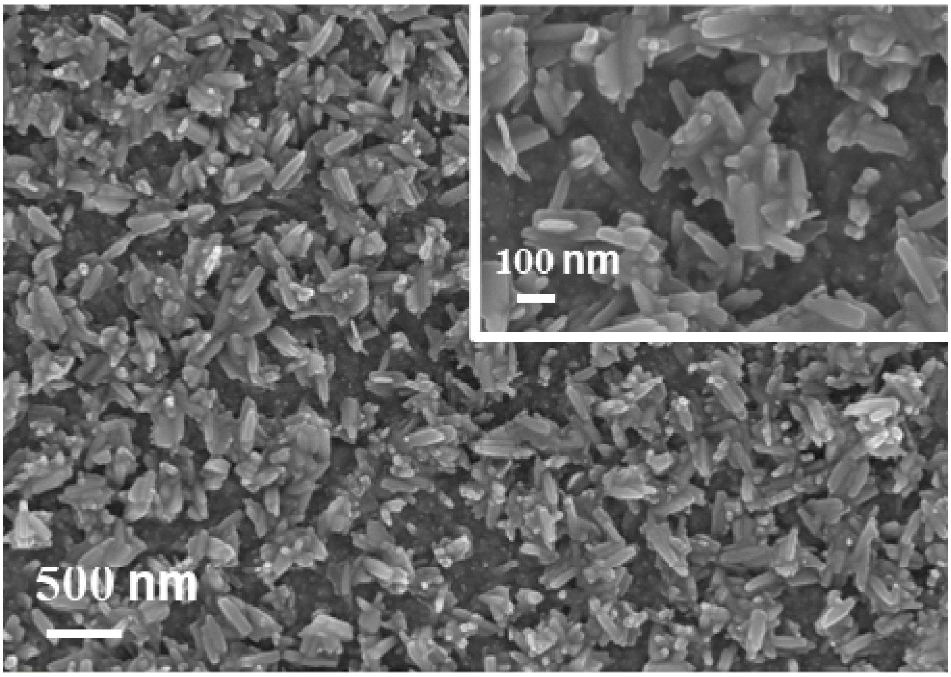

Figure S3 SEM image of the W-$M_{0.5}$ film

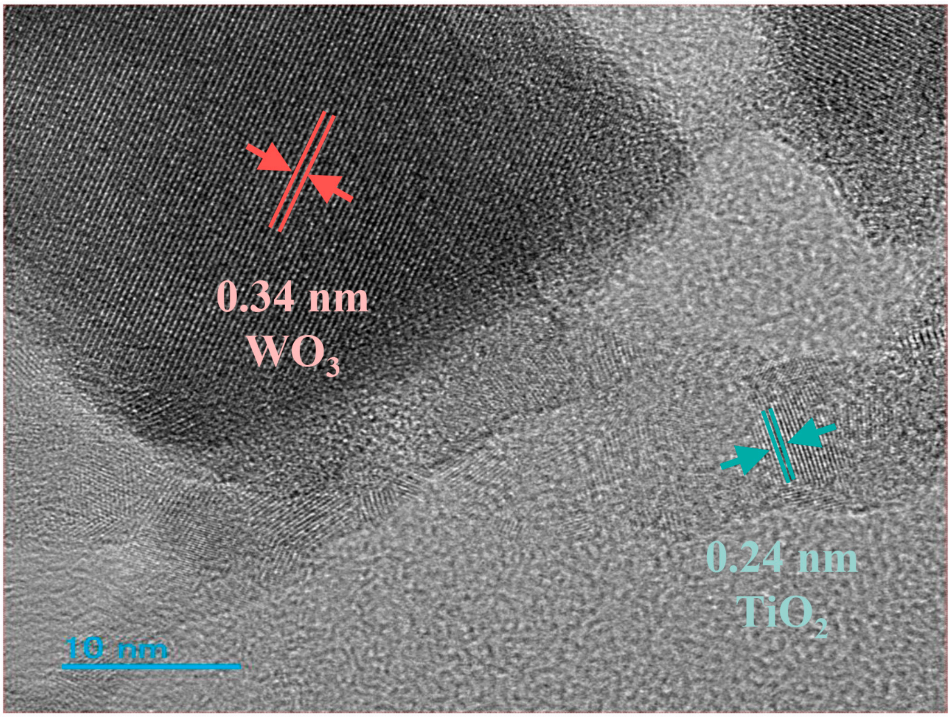

Figure S4 HR-TEM images of the W-$M_{1.0}$ films.

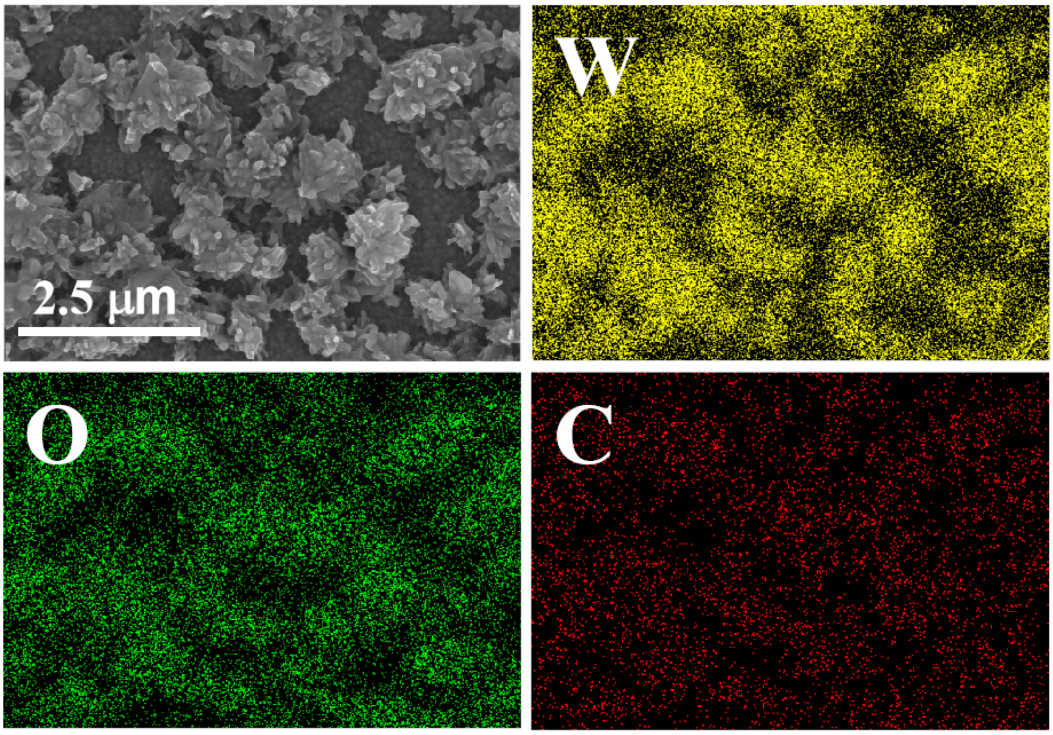

Figure S5 EDS-Mapping of the W-M_0 film

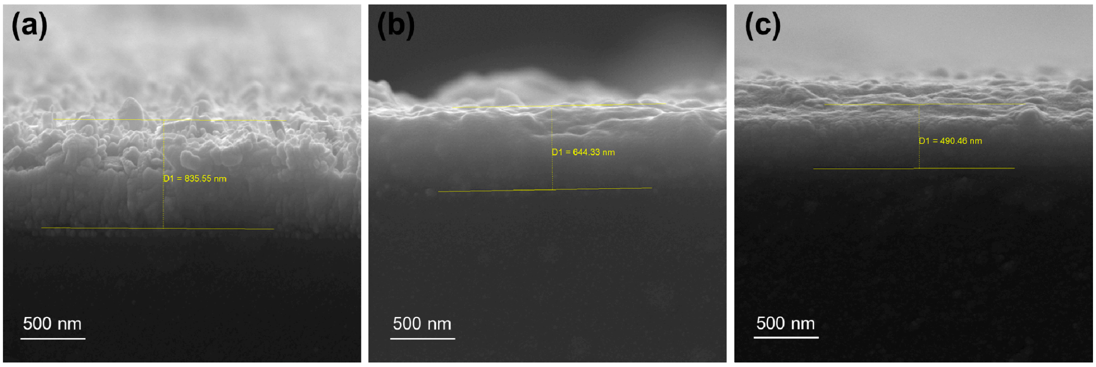

Figure S6 The thickness of (a) W-M_0 film, (b) W-$M_{0.5}$ film and W-$M_{1.0}$ film.

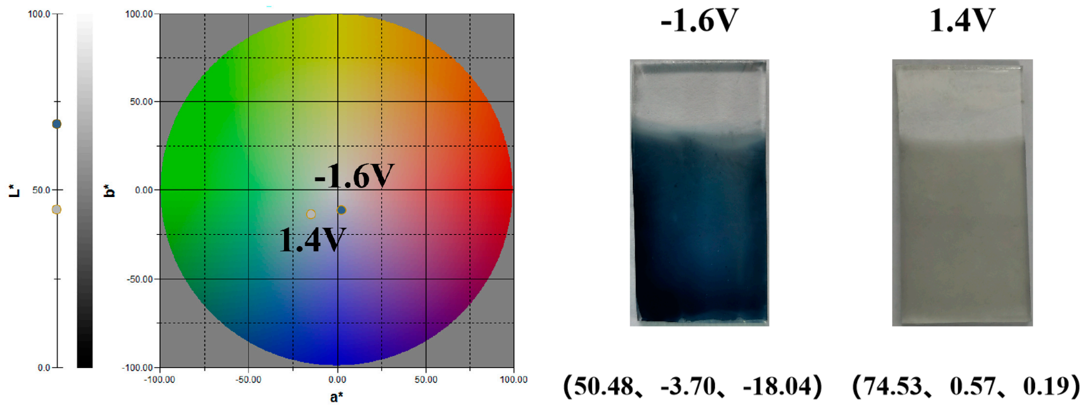

Figure S7 Optical photos and color values of W-M1.0 film under different voltages.

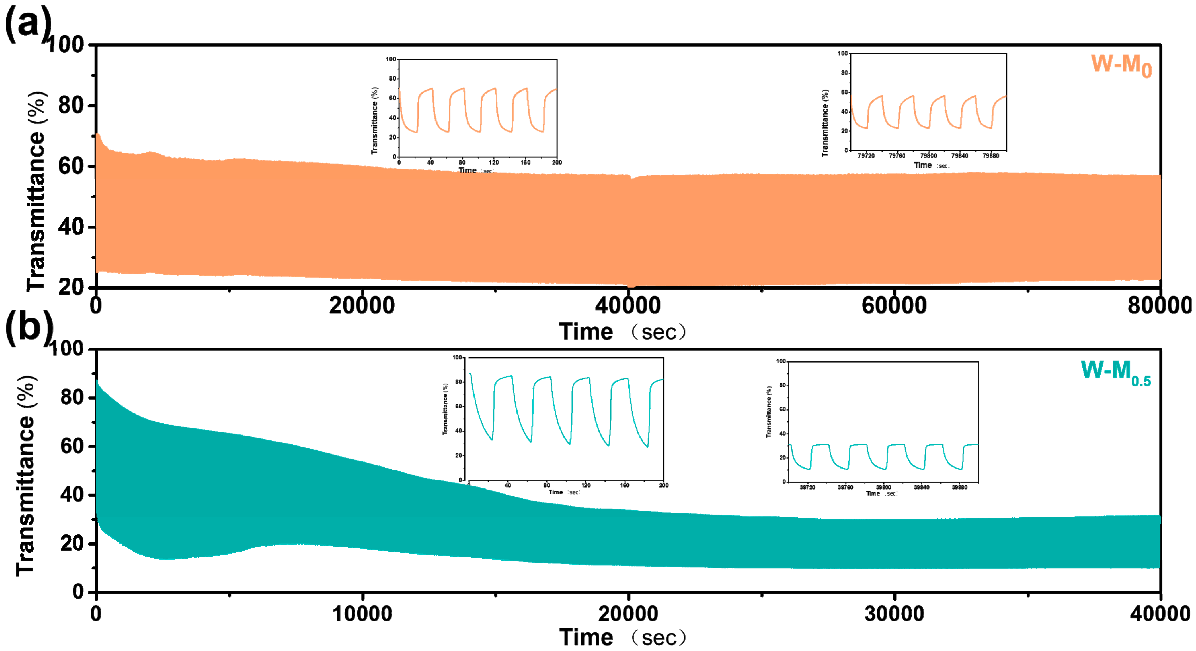

Figure S8 Long-term stability of (a) W-M0 films and (b) W-M0.5 films.

Table S1 Comparison of t_c, t_b, ΔT%, CE and cycling stability of W-$M_{1.0}$ films with other electrochromic materials.

	Wavelength (nm)	t_c (s)	t_b (s)	ΔT%	CE(cm^2 C^{-1})	Stability Data
self-doped WO_{3-x} films[36]	680	7.0	15	70	62	After 200 cycles, almost no degradation in CV curve
h@a-WNRAs films[37]	800	15	21	67.7	101.1	After 200 cycles, almost no degradation in CV curve
WO_3 films[38]	——	20.5	18.7	85	——	After 1500 cycles, 94% light transmittance modulation maintained
WMo-2 films[39]	600	15.2	12.9	81.40	100.81	After 1500 cycles, 94% light transmittance modulation maintained
ZnO/PEDOT[40]	550	1.8	2.0	51.4	128.95	After 8000 s of cycling, 84.2% light transmittance modulation maintained
P@C-dot-2[41]	——	11.8	8.8	——	——	After 2000 s of cycling, almost no degradation in optical modulation performance.
1% Ni-TiO_2[42]	——	12.8	1.99	32.1	——	After 200 cycles, almost no degradation in optical modulation performance.
This Work	1033	6.1	4.0	73.83	144.35	After 4000 cycles, 9.89% decay in light transmittance
